# Searching for the Haplorrhine Heterotherm: Field and Laboratory Data of Free-Ranging Tarsiers

**DOI:** 10.3389/fphys.2017.00745

**Published:** 2017-09-26

**Authors:** Shaun Welman, Andrew A. Tuen, Barry G. Lovegrove

**Affiliations:** ^1^School of Life Sciences, University of KwaZulu-Natal, Pietermaritzburg, South Africa; ^2^Institute of Biodiversity and Environmental Conservation, Universiti Malaysia Sarawak, Kota Samarahan, Malaysia

**Keywords:** metabolism, primate thermoregulation, tropics, evolution, tarsiers

## Abstract

The observation of heterothermy in a single suborder (Strepsirrhini) only within the primates is puzzling. Given that the placental-mammal ancestor was likely a heterotherm, we explored the potential for heterothermy in a primate closely related to the Strepsirrhini. Based upon phylogeny, body size and habitat stability since the Late Eocene, we selected western tarsiers (*Cephalopachus bancanus*) from the island of Borneo. Being the sister clade to Strepsirrhini and basal in Haplorrhini (monkeys and apes), we hypothesized that *C. bancanus* might have retained the heterothermic capacity observed in several small strepsirrhines. We measured resting metabolic rate, subcutaneous temperature, evaporative water loss and the percentage of heat dissipated through evaporation, at ambient temperatures between 22 and 35°C in fresh-caught wild animals (126.1 ± 2.4 g). We also measured core body temperatures in free-ranging animals. The thermoneutral zone was 25–30°C and the basal metabolic rate was 3.52 ± 0.06 W.kg^−1^ (0.65 ± 0.01 ml O_2_.g^−1^.h^−1^). There was no evidence of adaptive heterothermy in either the laboratory data or the free-ranging data. Instead, animals appeared to be cold sensitive (T_b_ ~ 31°C) at the lowest temperatures. We discuss possible reasons for the apparent lack of heterothermy in tarsiers, and identify putative heterotherms within Platyrrhini. We also document our concern for the vulnerability of *C. bancanus* to future temperature increases associated with global warming.

## Introduction

The capacity to become heterothermic conveys significant fitness benefits and promotes survivability (Geiser and Turbill, 2009; Geiser and Brigham, 2012; Lovegrove et al., 2014b; Nowack et al., 2015, 2016; Stawski et al., 2015; Lovegrove, 2017). These benefits may be derived either as a direct consequence of the reduction in energy expenditure and, in the case of hibernators specifically, the preservation of fat reserves during low resource availability (Lovegrove, 2000; Dausmann, 2014), or, indirectly by reducing the risk of predation due to decreased foraging effort (Bieber and Ruf, 2009; Stawski and Geiser, 2010; Bieber et al., 2014) as well as aiding in reproduction by manipulating foetal growth rate or by enhancing sperm storage (reviewed in Geiser and Brigham, 2012). However, whereas the benefits of heterothermy may be well documented, its origin remains hotly debated (Crompton et al., 1978; McNab, 1978; Bennett and Ruben, 1979; Hayes and Garland, 1995; Farmer, 2000; Koteja, 2000; Grigg et al., 2004; Kemp, 2006; Clarke and Pörtner, 2010; Lovegrove, 2012a,b, 2017).

With the aid of maximum likelihood character state reconstruction, Lovegrove (2012a) attempted to consolidate the literature and resolve the debate surrounding the antiquity of heterothermy relative to strict homeothermy in mammals. The study confirmed earlier work (see Grigg and Beard, 2000; Grigg et al., 2004) and showed that contrary to a long-standing paradigm, strict homeothermy is, as argued by Augee and Gooden (1992), the more derived state. This conclusion, in combination with recent work on brown antechinus (*Antechinus stuartii*; Stawski et al., 2015), sugar gliders (*Petaurus breviceps*; Nowack et al., 2015), and short-beaked echidnas (*Tachyglossus aculeatus*; Nowack et al., 2016), provided support for the argument that the ancestors of the three crown mammalian clades namely, the Monotremata, Marsupialia and Placentalia, likely survived the mass extinction event marking the Cretaceous-Paleogene (K-Pg) boundary because of their heterothermic capacity (Lovegrove et al., 2014b; Lovegrove, 2017).

For many decades, it appeared that heterothermy within the primates was geographically restricted to a single family—the Cheirogaleidae of Madagascar (see Dausmann, 2008). Now, in addition to observations of torpor and hibernation in Malagasy mouse lemurs (Ortmann et al., 1997; Aujard et al., 1998; Schmid and Kappeler, 1998; Schmid, 2000; Kobbe and Dausmann, 2009; Schmid and Ganzhorn, 2009) and hibernation in dwarf lemurs (Dausmann et al., 2000, 2005), heterothermy has been observed in two non-Malagasy primates. Torpor, despite the initial lack of evidence for it (Knox and Wright, 1989; Mzilikazi et al., 2006), occurs in the African lesser bushbaby (*Galago maholi*; Nowack et al., 2010), albeit under extreme conditions only. Hibernation has now also been observed in the pygmy slow loris in Vietnam (*Nycticebus pygmaeus*; Ruf et al., 2015). Even with the additional observations of heterothermy in the Galagidae and Lorisidae, all observations within Primates remain within the Strepsirrhini clade prompting the question why it has not also been observed in the haplorrhines? Has heterothermy potentially been “lost” in more derived primate clades or does its absence reflect an artefact of sampling bias?

In this paper, we sought to explore the potential for heterothermy in a non-strepsirrhine primate in an attempt to gain further insight into the primate heterothermy phenotype. Our choice of study animal was determined by three principal factors; (a) close phylogenetic relatedness to the strepsirrhines, (b) an insular tropical existence, and (c) small body size. Choosing a close relative provides the best opportunity to confirm a potential retention of the ancestral heterothermy condition. The island existence and small body size criteria stem from observations of extensive employment of heterothermy by small-bodied Malagasy lemurs and because most heterothermic mammals are small (<1 kg) (Geiser, 1998; Lovegrove, 2012a; Ruf and Geiser, 2015). It has been proposed that, in general, mammals that colonised tropical islands during the Early Cenozoic, that is, prior to the onset of global cooling during the Late Eocene, retained plesiomorphic climate-adaptation traits through stabilising selection (Hansen, 1997; Lovegrove, 2012a; Lobban et al., 2014).

Based on the aforementioned criteria, the most *apropos* model to search for the evidence of adaptive heterothermy in non-strepsirrhine primates is the tarsier (Tarsiidae). Tarsiers are small (80–150 g; but see Clarke, 1943), tropical, nocturnal and arboreal primates which inhabit the forests of South-east Asia (MacKinnon and MacKinnon, 1980; Crompton and Andau, 1986, 1987; Neri-Arboleda et al., 2002; Groves and Shekelle, 2010). The reported body temperatures (T_b_) of 33.3°C (Lovegrove et al., 2014a) and 33.8°C (McNab and Wright, 1987) for the Philippine tarsier (*Tarsius syrichta*) show that they are “basoendotherms” i.e., T_b_ < 35°C and thus similar to the predicted ancestral condition (*sensu* Lovegrove, 2012a); ca. 3°C lower than the average T_b_ of the primate clade (see Clarke et al., 2010). Since their evolution during the Eocene, tarsiers have persisted within a continuously tropical environment in habitats that are argued to be very similar to those that their ancestors inhabited; despite some changes in floristic composition (Jablonski, 2003; Simons, 2003). They are also the only strictly carnivorous primate and, as a rule, take live prey (Jablonski and Crompton, 1994; Gursky, 2000). With regards to their phylogeny, their position has been hotly debated and shuffled around the primate tree (Schwartz, 1984; Schmitz et al., 2001; Meireles et al., 2003; Simons, 2003; Yoder, 2003; Matsui et al., 2009; Perelman et al., 2011). However, the most recent study by Hartig et al. (2013) supports the Haplorrhini hypothesis i.e., that tarsiers are the sister taxa to the Anthropoids, as originally proposed by Pocock (1918). Tarsiers are thus the closest extant relatives to the strepsirrhines.

Given the close phylogenetic relationship of tarsiers to the Strepsirrhini, as well as the varied observations of torpor in the Strepsirrhini (Dausmann, 2014; Dausmann and Warnecke, 2016), we predicted that stabilizing selection may have favoured the retention of the plesiomorphic capacity for heterothermy within tarsiers (Lovegrove, 2012a). Currently, there is no physiological evidence in tarsiers that supports our prediction. Most of our understanding of tarsier thermoregulation does however stem from three studies on *T. syrichta* (Clarke, 1943; McNab and Wright, 1987; Lovegrove et al., 2014a), but it has been speculated that western tarsiers (*Cephalopachus bancanus*, previously *T. bancanus*) may be capable of torpor (Niemitz, 1984; Niemitz et al., 1984).

Our study had two main objectives. The first was to determine the thermoregulatory response of wild-caught *C. bancanus* to varying ambient temperatures (T_a_), noting any potential indication of hypometabolism or the associated reduction in T_b_. The second was to document free-ranging core temperatures (T_core_) continuously over several months to determine whether torpor occurs in their natural setting.

## Materials and methods

### Animal capture and husbandry

Nine male and four non-pregnant female adult tarsiers were used in the study. Animals were captured in mist nets during two sampling periods between August–October 2014 and March–August 2015, at Sama Jaya Nature Reserve (1°31′16″ N; 110°23′15″ E) in Kuching City, Sarawak, on the island of Borneo. The vegetation type of the study area was secondary forest and the reserve encompassed an area of approximately 38 hectares. The number of daylight hours remained fairly constant, with sunrise typically occurring shortly after 06:00 and sunset occurring shortly before 19:00. Nets were set in areas with a dense concentration of narrow-stemmed trees, as well as in areas with notable olfactory cues from scent markings of resident tarsiers. All nets were opened at dusk (ca. 18:00) and checked at regular intervals throughout the night during the tarsier's active phase (α-phase). Captured individuals were sexed and weighed using an electronic scale. Temperature-sensitive passive integrated transponder (PIT) tags (Biomark HDX12, Boise, Idaho, USA) were injected into their flanks. The PIT tags enabled us to measure the animals' subcutaneous body temperatures (T_sub_) and also provided a unique identification code. The PIT tag's location was chosen in order to avoid any potential harm to their vital organs, while still being situated in close proximity to their core region during normal posture. After the injection, the animals were rehydrated and housed in a covered wire mesh cage fitted with branches. During captivity, animals were fed live lizards or crickets and provided with water *ad libitum*. However, to ensure a post-absorptive status during measurements, all animals received their last meal 6 h prior to respirometry measurements. Tarsiers are notoriously difficult to maintain in captivity so all animals were held captive for a maximum of 36 h only.

All experimental procedures were reported to and approved by the University of Kwazulu-Natal Animal Ethics Committee (116/13/Animal), which adopts the guidelines described by the Canadian Council on Animal Care. All experimental protocols were also approved by the Sarawak Forestry Department [permit number: NCCD.907.4.4(9)-223, NCCD.907.4.4(13)-277].

### Gas exchange measurements

We used the incurrent flow measurement flow-through respirometry design described in Lighton (2008) to measure the rate of oxygen consumption ($\dot{V}O_{2}$), carbon dioxide production ($\dot{V}CO_{2}$), and evaporative water loss (EWL) of tarsiers exposed to varying T_a_s. Animals were housed in sealed 4l respirometers that were constructed from clear plexiglass acrylic. Respirometers were fitted with a grid platform elevated above a 1 cm deep layer of mineral oil used to trap urine and faeces. Dried and CO_2_-free air was flowed through the chamber at constant rates between 400 and 500 ml.min^−1^, sufficient to maintain O_2_ concentrations of 20.8–20.0% within the respirometer. Incurrent air was dried and scrubbed of CO_2_ by drawing it through a PC-4 Condensing Dryer (Sable Systems, Las Vegas, USA), followed by a column of silica gel and a column of indicating soda lime, and finally a column of indicating Drierite before reaching the pump and flow meter unit (SS-4 sub-sampler, Sable Systems). With the aid of a RM-8 Flow Multiplexer (Sable Systems), the excurrent air from the animal chamber and a stream of reference air were sequentially subsampled and passed through a series of gas analysers. The water vapour content of the air was measured using a RH-300 water vapour analyser (Sable Systems), whereafter it was dried again and flowed through a field gas analysis system (Foxbox-C, Sable Systems) to measure the fractional concentrations of CO_2_ and O_2_. CO_2_ was scrubbed from the air stream prior to the O_2_ analyser. Sable System's data acquisition software, Expedata (v 1.7.22), was used to interpret and record the digital outputs from the equipment using a laptop at 1-s intervals. The respective rates of EWL, $\dot{V}CO_{2}$, and $\dot{V}O_{2}$ were calculated using the equations presented in Withers (2001).

The O_2_ analyser was spanned prior to every measurement and the water vapour and CO_2_ gas analysers were calibrated monthly. Compressed pure nitrogen gas was used to set the zero point during all calibrations. A bubbler flask and waterbath were used to generate humid air of a known dew point in order to set the water vapour span values. The CO_2_ span value was set using certified commercially available compressed CO_2_ gas.

### Experimental protocol for gaseous exchange measurements

We determined the animals' thermoregulatory response by concurrently measuring resting metabolic rate (RMR) and T_sub_ adjustments while exposed to temperatures between 22 and 35°C. The air temperature within the respirometers was measured using commercially available temperature sensitive data-loggers (iButton DS1922L, Thermochron, Dallas, TX, USA; resolution: 0.0625°C), hereafter temperature loggers. The experimental temperatures were controlled by partially submerging the respirometers into a body of water within a modified coolerbox, wherein the temperature of the water was regulated using a waterbath via a closed loop, such that the water flowed from the waterbath into the coolerbox and back into the waterbath. A Biomark HPR plus reader and antennae were used to read and record T_sub_ every 30 s; providing a real-time visualization of the animals' thermal profile during measurements. Temperature loggers and PIT tags were calibrated following the method of Cory Toussaint and McKechnie (2012).

All measurements were performed on solitary individuals during their rest phase (ρ-phase; 06:00–18:00). Each tarsier was exposed to a maximum of three temperatures per measurement, in a random order, for 3–6 h to allow adequate time for thermal adjustment. There were two instances where measurements were terminated prematurely because the individuals remained restless and their T_sub_s increased rapidly to 38°C; both occurred at T_a_ = 35°C. Data from these two individuals were excluded from analyses.

### Surgical procedure and free-ranging body temperature measurements

After the completion of respirometery measurements, five of the 13 animals (three males and two females) used in the respirometry study, were surgically implanted with the same custom designed temperature sensitive data-loggers (MCP 9800, Microchip Technology, Chandler, AZ; resolution: 0.0625°C) described in Lovegrove et al. (2014a) to measure T_core_ in free-ranging animals. The loggers were assembled on site at our research station at the Universiti Malaysia Sarawak. A 3-volt CR 1632 coin battery was soldered to the terminals of the logger circuit board and the units were then coated with an acrylic protective lacquer (Electrolube, HK Wentworth Ltd., Leicestershire, UK) and encapsulated in surgical wax; yielding a final weight of ca. 4g. In compliance with the conditions stipulated for the approval of permits, all surgical procedures were performed by a local veterinarian (Dr. Samuel Kiyui, Malaysian Veterinary Council registration number: 049) under sterile conditions at his surgery. Anaesthesia was induced by a 1ml intramuscular injection of a cocktail containing Tiletamine and Zolazepam as active ingredients (Zoletil 100, Virbac Veterinary Pharmaceutics), whereafter the data loggers were inserted into the peritoneal cavity via an incision along the *linea alba*. The incision was sutured using 3/0 absorbable Dexon polyglycolic acid suture and a topical antiseptic [Chlorhexidine gluconate 5% (w/v) and Isopropyl alcohol 3.15% (w/v)] was liberally applied to the area. While anaesthetized, animals were also fitted with external VHF radio-telemetry transmitters (PD-2C, Holohil Systems Ltd., Ottawa, Canada; weight = 4 g) to allow us to track and monitor the animals. Tarsiers were released at their site of capture as soon as possible once they had regained their mental acuity and monitored for the first hour following release. Thereafter, daily observations were made for the first week and then monitoring became periodical to minimize disturbance.

The T_core_ data loggers were calibrated in a similar manner to that previously mentioned. They were programmed to record readings every 30 min for a period of 6 months. Of the five implants, we recovered 3 and 5 weeks of data from a male and female, respectively. The unsuccessful attempts were due to premature battery failure caused by the soldering process.

The T_a_ within the forest was recorded throughout the study period using the same type of temperature loggers that were used during metabolic measurements. The loggers were housed in black and white solar radiation shields that were attached as a pair (1 black and 1 white) at a height of 1.5m above the ground to three trees located in different sections of the reserve. All three trees were observed tarsier rest sites.

### Data analyses

#### Laboratory measurements

A lag correction was performed in Expedata in order to synchronize the respective gas-exchange traces before any calculations were made. RMR and EWL were calculated from steady-state traces corresponding to the most level continuous 10-min section of the $\dot{V}O_{2}$ trace identified using Expedata functions. The accompanying body mass (M_b_) was calculated using a regression of the animals' M_b_ at the start and end of metabolic measurements. Metabolic rate (in Watts) was calculated by converting the respiratory exchange ratio ($\dot{V}CO_{2}$/$\dot{V}O_{2}$) using the thermal equivalence data in Table 4.2 in Withers (1992). Evaporative heat loss (in Watts) was calculated assuming 2.26 J. mg H_2_O^−1^, and then used to calculate the amount of metabolic heat dissipated through evaporation as the ratio of evaporative heat loss to metabolic heat production (EHL/MHP).

In order to objectively identify inflection points along the profiles of RMR, T_sub_, EWL and EHL/MHP as a function of T_a_, we performed a piecewise regression analysis in R 3.0.2 (R Core Team, 2017) using the package “segmented” (Muggeo, 2008). Each section of data identified through the analysis was treated as independent and we adopted the approach of implementing mixed-effect models using the R package “nlme” (Pinheiro et al., 2016) to evaluate the effect of T_a_ while accounting for other variables. We developed models using various permutations of M_b_ and sex as fixed factors. To account for repeated measurements in laboratory measurements (*n* = 13) we included individual ID as a random factor in all of the models. We then compared their Akaike Information Criterion values that were corrected for small sample size (AICc and AICcWt; Burnham and Anderson, 2003) to determine the model of best fit using the R package “AICcmodavg” (Mazerolle, 2015). We tested for a gender difference in M_b_ at the time of first capture using a Student's *t*-test. All values are reported as mean ± standard error unless stated otherwise. The assumptions of the models were verified in a similar manner to Levesque et al. (2014).

#### Free-ranging measurements

We used Rayleigh's tests to assess whether there was any uniformity in the times at which maximum and minimum T_core_ in free-ranging animals (*n* = 2) and forest temperatures were observed. We also tested whether T_core_ followed a normally distributed pattern, using a Shapiro-Wilk's test.

## Results

### Thermoregulatory measurements

All thermoregulatory measurements represent complete sets of physiological variables obtained concurrently during laboratory experiments using 13 wild-caught animals. The mean M_b_ at first capture was 126.1 ± 2.4 g. Males (*n* = 9, M_b_ = 126.9 ± 2.7 g) were significantly heavier than females [*n* = 4, M_b_ = 117.1 ± 2.7 g; one-tailed *t*_(11)_ = 2.76, *p* = 0.009].

**Figure 1:**
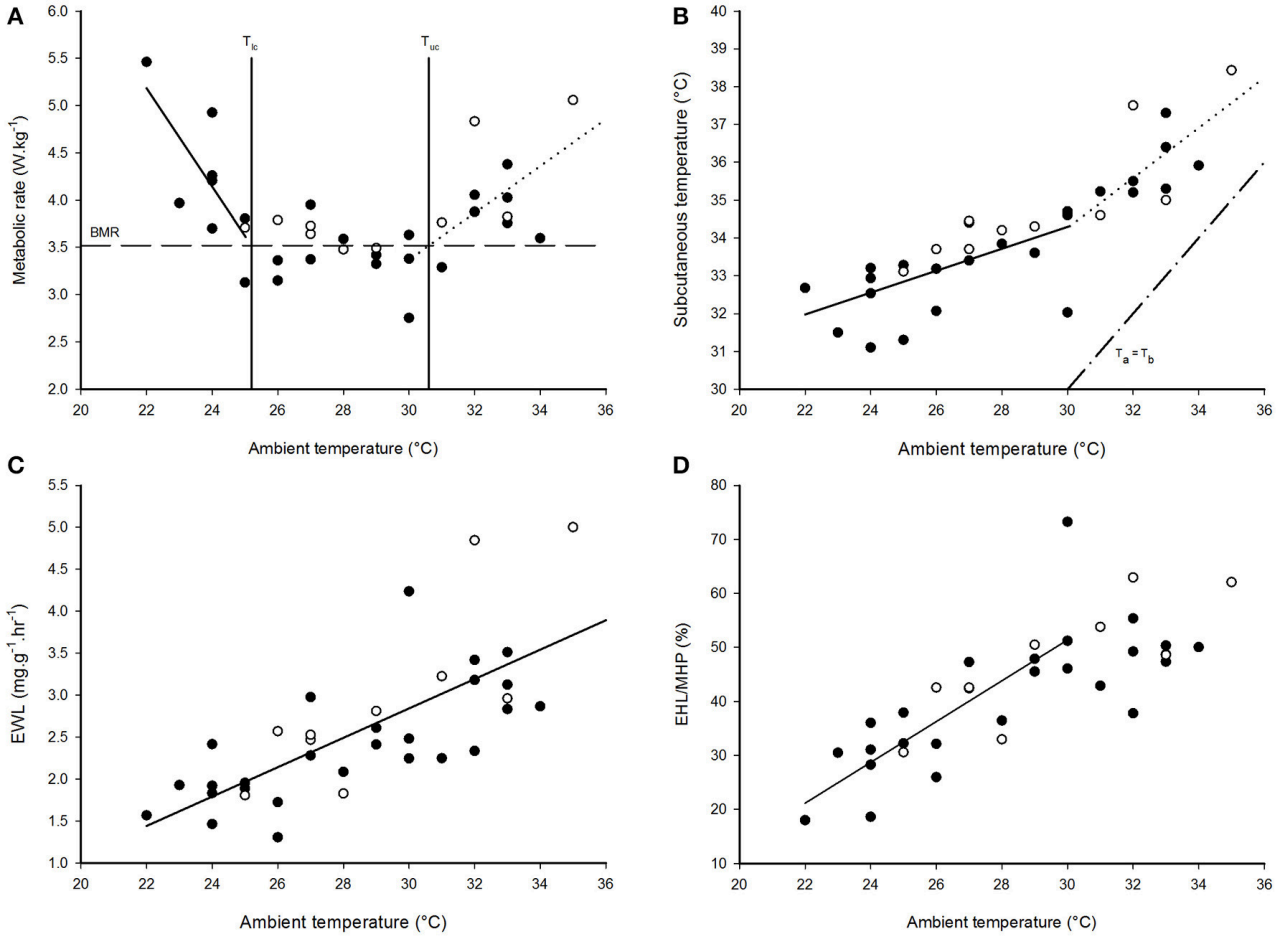
The thermoregulatory profile of *Cephalopachus bancanus*. **(A)** Effect of ambient temperature on resting metabolic rate. **(B)** Effect of ambient temperature on subcutaneous temperature. **(C)** Effect of ambient temperature on the rate of evaporative water loss. **(D)** Effect of ambient temperature on the amount of metabolic heat dissipated through evaporative cooling. Plotted regression lines indicate the best fit for the data based on piecewise regression analysis and the equations are presented in Table 1. Closed circles = males, open circles = females.

Mass-specific RMR displayed inflection points at T_a_ = 25.0 ± 1.3°C (±95% confidence interval; CI) and T_a_ = 30.0 ± 2.5°C (±95% CI), and displayed a significant and linear relationship with T_a_s < 25°C [*F*_(1, 7)_ = 9.44, *p* < 0.018, *r*^2^ = 0.57] and T_a_s > 30°C [*F*_(1, 13)_ = 9.99, *p* = 0.007, *r*^2^ = 0.43], but not with T_a_s between these values (Figure 1A, Table 1). Thus, the species' thermoneutral zone (TNZ) was ca. 25–30°C. We calculated a basal metabolic rate (BMR) of 3.52 ± 0.06 W.kg^−1^ (0.65 ± 0.01 ml O_2_.g^−1^.h^−1^) by averaging all RMR values between and excluding those at the inflection points; including the values from both inflection points made no difference. The accuracy of the thermal limits proposed by the piecewise regression analysis was confirmed by substituting BMR into the respective regression equation of each line, in order to calculate the T_a_ at which they intersected BMR. The intersection corresponding to the lower limit of thermoneutrality (T_lc_) was at T_a_ = 25.2°C and the upper limit (T_uc_) was at T_a_ = 30.6°C. The models which best predicted whole-animal RMR above (Akaike weight = 0.74) and below (Akaike weight = 0.70) TNZ included T_a_ as the only fixed factor (Table 2). In both cases, the next best models contained M_b_ as an additional factor, and explained all of the remaining Akaike weight for metabolism below TNZ. The remaining Akaike weight for metabolism above TNZ included gender as a factor.

**Table 1:** Linear regression models describing the relationship between ambient temperature (T_a_) and various physiological parameters in *Cephalopachus bancanus*.

**Parameter**	**Temperature range**	**Regression equation**
RMR	T_a_ < 25°C:	RMR = 16.65 − 0.5212 × T_a_^*^
	T_a_ > 30°C:	RMR = 0.2479 × T_a_ − 4.069^**^
		
T_sub_	T_a_ < 30°C:	T_sub_ = 25.64 + 0.2879 × T_a_^*^
	T_a_ > 30°C:	T_sub_ = 14.64 + 0.6547 × T_a_^*^
		
EWL	All	EWL = 0.17512 × T_a_ − 2.41254^*^
		
EHL/MHP	T_a_ < 30°C:	EHL/MHP = 3.761 × T_a_ − 61.52^*^

*Mass-specific resting metabolic rate = RMR, subcutaneous temperature = T_sub_, the rate of evaporative water loss = EWL, the amount of metabolic heat dissipated through evaporative cooling = EHL/MHP*,

* *p < 0.05*,

** *p < 0.01*.

**Table 2:** Linear mixed-effect models which best describe the significant relationships between ambient temperature (T_a_) and resting metabolic rate (RMR), subcutaneous temperature (T_sub_), the rate of evaporative water loss (EWL) and the amount of metabolic heat dissipated through evaporative cooling (EHL/MHP) in *Cephalopachus bancanus*.

**Fixed factors**	**Random factor**	***k***	**AICc**	**Akaike weights**
**RMR (T**_a_ > **30**°**C)**				
T_a_	~1|ID	4	−37.92	0.74
T_a_ + M_b_	~1|ID	5	−34.89	0.16
T_a_ + Sex	~1|ID	5	−33.34	0.07
T_a_ + M_b_ + Sex	~1|ID	6	−31.08	0.02
**RMR (T**_a_ < **25**°**C)**				
T_a_	~1|ID	4	−0.69	0.7
T_a_ + M_b_	~1|ID	5	1.01	0.3
**T**_sub_ **(T**_a_ > **30**°**C)**				
T_a_	~1|ID	4	51.57	0.82
T_a_ + Sex	~1|ID	5	56.01	0.09
T_a_ + M_b_	~1|ID	5	56.15	0.08
T_a_ + M_b_ + Sex	~1|ID	6	61.34	0.01
**T**_sub_ **(T**_a_ < **30**°**C)**				
T_a_ + M_b_ + Sex	~1|ID	6	43.15	0.73
T_a_ + M_b_	~1|ID	5	45.81	0.19
T_a_	~1|ID	4	48.06	0.06
T_a_ + Sex	~1|ID	5	50.88	0.02
**EWL**				
T_a_	~1|ID	4	117.26	0.58
T_a_ + Sex	~1|ID	5	119.66	0.17
T_a_ + M_b_	~1|ID	5	119.67	0.17
T_a_ + M_b_ + Sex	~1|ID	6	121.47	0.07
**EHL/MHP (T**_a_ < **30**°**C)**				
T_a_	~1|ID	4	−52.66	0.65
T_a_ + M_b_	~1|ID	5	−50.19	0.19
T_a_ + Sex	~1|ID	5	−49.62	0.14
T_a_ + M_b_ + Sex	~1|ID	6	−46.19	0.03

*Only models with Akaike weights ≥ 0.01 are presented*.

T_sub_ displayed a single inflection point during the thermoregulatory measurements coincident with T_uc_ (Figure 1B). Both sections of the T_sub_ profile had a significant and linear relationship with T_a_ [T_a_ < 30°C: *F*_(1, 19)_ = 19.58, *p* < 0.001; T_a_ > 30°C: *F*_(1, 13)_ = 15.68, *p* = 0.002], but the slope of the relationship for T_a_ < 30°C was lower (Table 1). The model which best predicted T_sub_ above T_uc_ included T_a_ as the only fixed factor (Akaike weight = 0.82), whereas the model best suited to predict T_sub_ below T_uc_ included all of the variables (Akaike weight = 0.73). The mean minimum T_sub_ was 32.4 ± 0.5°C observed at T_a_ = 24°C, whereas at higher T_a_s there was no difference in the mean maximum T_sub_ observed at T_a_ = 32°C (T_sub_ = 35.9 ± 0.5°C) and T_a_ = 33°C (T_sub_ = 36.0 ± 0.5°C).

EWL had a significant, positive linear relationship with T_a_ [*F*_(1, 34)_ = 37.9, *p* < 0.001, *r*^2^ = 0.53, Figure 1C, Table 1] and was best predicted by a model containing T_a_ as the only fixed factor (Akaike weight = 0.58, Table 2). EWL ranged from a mean minimum of 1.86 ± 0.37 mg^−1^.g^−1^.h^−1^ at T_a_ = 26°C, to a mean maximum of 3.44 ± 0.52 mg^−1^.g^−1^.h^−1^ at T_a_ = 32°C. Barring the elevated rate at T_a_ = 27°C, EWL remained < 2 mg^−1^.g^−1^.h^−1^ and was stable at T_a_s ≥ 28°C. In addition, EWL displayed a strong correlation with T_sub_ (*r* = 0.81), and T_sub_ was correlated to the amount of EHL/MHP (*r* = 0.67).

The percentage of EHL/MHP scaled linearly with T_a_ below T_uc_ [*F*_(1, 19)_ = 29.7, *p* < 0.001, Figure 1D, Table 1]. At T_a_s > 30°C, EHL/MHP remained relatively constant and no longer displayed any relationship with T_a_. The mean maximum percentage of EHL/MHP was 57 ± 6% and was achieved at T_a_ = 30°C. The model which best predicted EHL/MHP below T_uc_ was again the one which included T_a_ as the only fixed factor (Akaike weight = 0.65).

### Free-ranging body temperature profile

Both tarsiers were implanted on the 19th June 2015, but we excluded all values prior to the 24th of June to minimize any potentially misleading data recorded during the period of recuperation.

**Figure 2:**
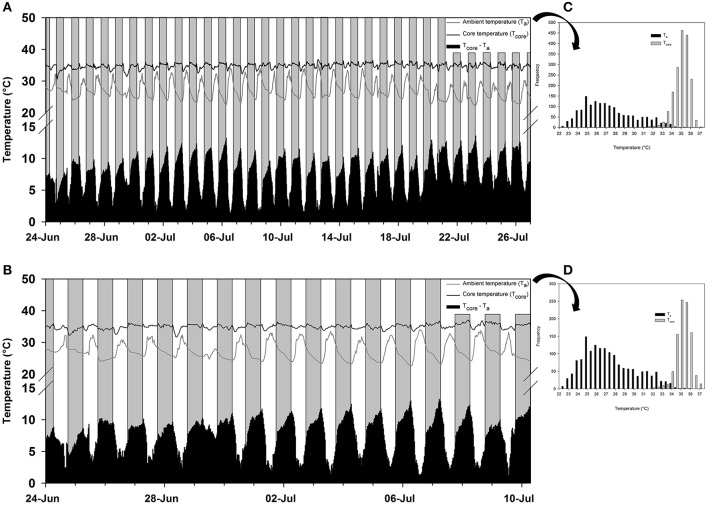
The free-ranging temperature profile in western tarsiers (*Cephalopachus bancanus*). **(A)** The ambient temperatures (T_a_; gray line) and core temperatures (T_core_; black line) recorded in a female from 00:00 24th June 2015 to 00:00 27th July 2015. **(B)** The ambient temperatures (T_a_; gray line) and core temperatures (T_core_; black line) recorded in a male from 00:00 24th June 2015 to 06:30 10th July 2015. The grey and white bars represent night and day, respectively, and the black bars at the base of each plot represent the temperature differential between the T_a_ and T_core_. **(C,D)** The frequency distribution of the respective T_a_s and T_core_s observed in the female and male tarsier's temperature profile.

**Figure 3:**
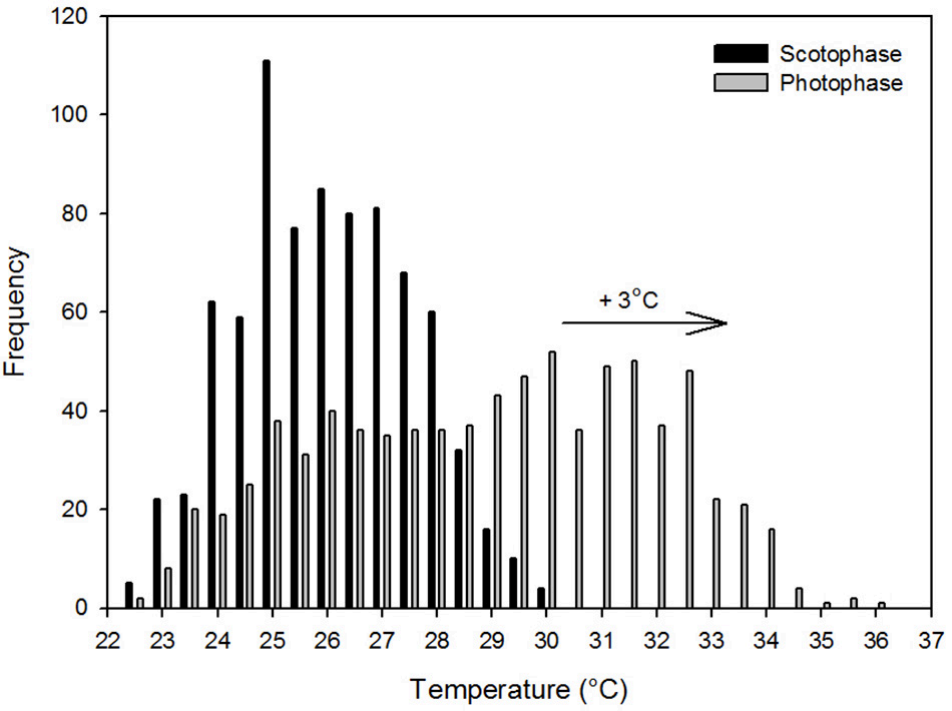
The frequency distribution of the scotophase and photophase ambient temperatures recorded within the forest at Sama Jaya Nature Reserve from 00:00 24th June 2015 to 00:00 27th July 2015. The arrow indicates the commonly predicted shift of three degree Celsius in the modal temperature associated with global warming.

The frequency distribution (0.5°C incremental bin category) of T_core_ did not conform to a Gaussian distribution pattern (♂: *W* = 0.98, *p* < 0.001; ♀: *W* = 0.96, *p* < 0.001), but displayed a slightly left-skewed unimodal pattern. For both individuals, the modal T_core_ was 35.0 ± 0.5°C (Figures 2C,D) and only once, in the male, did T_core_ exceed 37°C. Quantile 1 of the free-ranging T_core_s were similar between individuals (Q1: ♂ = 34.5°C vs. Q1: ♀ = 34.3°C) but the female displayed a higher propensity for T_core_ to decrease below Q1 (♀: 16% of all T_core_s vs. ♂: 8% of all T_core_s). The mean daily range in T_core_ was 2.4°C for both individuals and the times at which the maximum and minimum T_a_s and T_core_s (excluding the maximum T_core_ of the male) were observed were not uniformly distributed throughout a 24-h period (Rayleigh's test, *p* < 0.001; Table 3). Peak T_core_s during the α-phase occurred consistently at 19:00 or 06:00 which coincided with the highest and lowest scotophase T_a_s, respectively. The lowest T_core_s were consistently observed at or before 10:00, preceding the onset of the rapid increase in T_a_ to the daily maximum (range: 27.9–35.2°C, Table 3) at ca. 14:30. The frequency distribution of T_a_s during the scotophase were slightly right skewed and unimodal, with a peak in frequency at T_a_ = 25°C (Figure 3). The frequency distribution of T_a_s during the photophase were left skewed and had a flat-shaped distribution; the highest frequencies were observed between T_a_ = 30.0–32.5°C (Figure 3). The T_core_-T_a_ gradient was lowest during the ρ-phase and was ≤3°C on 75 and 70% of the observation days for the male and female, respectively; of those, 42 and 61% were ≤2°C (Figures 2A,B).

**Table 3:** The daily mean, maximum and minimum ambient (T_a_) and tarsier core (T_core_) temperatures observed throughout the period of free-ranging data collection (values in parenthesis represent the range in daily means), as well as the times at which these parameters where most frequently observed.

	**Photophase (**ρ**-phase)**			**Scotophase (**α**-phase)**		
	**T_a_ (*n* = 33)**	**♀ T_core_ (*n* = 33)**	**♂ T_core_(*n* = 16)**	**T_a_ (*n* = 33)**	**♀ T_core_ (*n* = 33)**	**♂ T_core_ (*n* = 17)**
Mean	28.6°C (25.75–31.0°C)	34.4°C (32.9–35.2°C)	34.7°C (33.9–35.6°C)	25.8°C (23.8–27.7°C)	35.1°C (33.5–35.7°C)	35.3°C (34.1–36.4°C)
Maximum	32.1°C (28.0–35.2°C)	35.8 (34.9–36.8°C)	35.7°C (35.0–36.9°C)	28.2°C (24.3–29.9°C)	35.8°C (34.0–36.4°C)	36.1°C (37.1–35.3°C)
Minimum	24.3°C (22.3–27.0°C)	33.4°C (31.5–34.4°C)	33.7°C (31.6–35.3°C)	24.30°C (22.2–27.1°C)	34.4°C (30.6–35.3°C)	34.4°C (32.2–35.8°C)
	**The time of the day at which maximum and minimum temperatures occurred and the relative percentage of observations**					
	**T**_a_ **max**	**T**_a_ **min**	♀ **T**_core_ **max**	♀ **T**_core_ **min**	♂ **T**_core_ **max**	♂ **T**_core_ **min**
Photophase (ρ-phase)	^†^13:00 (12%); 13:30 (15%); 14:00 (24%); 14:30 (18 %)	^†^06:30 (15%); 07:00 (61%)	^†^06:30 (76%)	^†^08:30 (15%); 09:00 (21%); 09:30 (30%)	06:30 (53%); 14:30 (12%); 16:00 (18%)	^†^08:30 (12%); 09:00 (24%); 09:30 (24%); 10:00 (12%); 18:00 (12%)
Scotophase (α-phase)	^†^00:30 (12%); 18:30 (82%)	^†^05:30 (24%); 06:00 (45%)	^†^06:00 (18%); 19:00 (42%)	^†^02:00 (12%); 04:00 (12%); 04:30 (15%); 18:30 (27%)	06:00 (18%); 19:00 (59%)	^†^00:00 (18%); 00:30 (12%); 01:00 (12%); 03:30 (18%);

Photophase corresponds to the animals rest phase, whereas scotophase corresponds to their active phase. ♂, male; ♀, female; max, highest temperature; min, lowest temperature; n, periods of observations and

† *indicates a significant (p < 0.05) Rayleigh's test*.

## Discussion

Our study provides no clear indication of metabolic suppression or the characteristic concomitant decrease in body temperature associated with heterothermy in either wild-caught animals measured in the laboratory or in free-ranging individuals. For the field data, having a single individual per sex limits our deductions regarding the finer details and interpretation of the species' free-ranging T_core_ pattern. Nonetheless, we are able to provide a few key observations and relate them to data from the laboratory.

The lowest temperatures to which tarsiers were exposed, both in the laboratory and in the field, was approximately 20°C, that is, about 5°C lower than the T_lc_. Typically, it is at temperatures <T_lc_ that heterotherms enter daily torpor or hibernation. Instead, tarsiers appeared to show cold sensitivity. During laboratory measurements, it became increasingly difficult for the animals to maintain T_b_s > 33°C at T_a_s < 30°C despite their best thermoregulatory attempts at metabolic upregulation. The decreasing trend in T_b_ at low temperatures suggests that the animals may have been heading towards mild pathological hypothermia at the lowest ambient temperatures. One observation in the free-ranging data provides a margin of support for our argument. T_core_ in the female decreased to 31.5°C during the afternoon on the 29th of June (Figure 2A) following a cold snap and only rebounded after T_a_ started to increase later during the day. However, the male did not display the same response during the cold snap. That tarsiers may be acutely cold sensitive does not seem far-fetched considering that they evolved in a warm tropical climate and have never left those conditions (Jablonski, 2003; Simons, 2003).

In contrast to the body temperature profile observed in free-ranging *T. syrichta* individuals on Bohol Island in the Phillipines (Lovegrove et al., 2014a), free-ranging *C. bancanus* maintained higher T_core_s during their active phase. In that study, *T. syrichta* displayed an extremely unusual pattern for a nocturnal mammal (Aschoff, 1983; Refinetti and Menaker, 1992) with consistent and considerable heat storage occurring during the photophase. Heat storage, barring one exceptionally hot day where T_core_ was elevated to T_core_ = 36.8°C (see July 13, Figure 2B), did not occur routinely during the photophase in this study. Furthermore, *C. bancanus* maintained a range of T_core_s that bordered the boundary between basoendothermy and “mesoendothermy” (35°C ≤ T_b_ ≤ 37.9°C) (*sensu* Lovegrove, 2012a), but were more frequently representative of mesoendothermic values. Thus, whereas *T. syrichta* appeared to be a strict basoendotherm (Lovegrove et al., 2014a), *C. bancanus* maintained normothermic T_core_s which were closer to the average T_b_ of the primate clade (see Clarke et al., 2010). The free-ranging T_core_ in our study suggests that tarsiers are intermediate between the ancestral low T_b_ condition and the derived higher T_b_ condition (Lovegrove, 2012a), which supports the phylogenetic position of tarsiers as the link between the older and derived primates (Hartig et al., 2013). Notably, the data from *T. syrichta* were skin temperatures (T_skin_) measured as a proxy for core temperatures; a technique which is susceptible to unreliable measures (Dausmann, 2012; Lovegrove et al., 2014a) especially, as we suspect is the case in the Bohol study, by loose-fitting collars. For now, heat storage may not be as prominent in tarsiers as the Bohol data suggested, but this may change within the near future.

It has been argued that the temperature increase associated with global warming will have a mild effect on *T. syrichta*, in spite of their T_skin_ pattern, because their TNZ was above the maximum T_a_s observed in their habitat (McNab and Wright, 1987; Lovegrove et al., 2014a). The same notion does not apply here. The TNZ range of *C. bancanus* (Figure 1A), is approximately 5°C lower than that of *T. syrichta*. A conservative prediction of a 3°C increase in T_a_s, relative to the modal T_a_s observed in our study, would result in a large proportion of the photophase T_a_s shifting above the TNZ of *C. bancanus* (see Figure 3). Offloading excess body heat as T_a_ approaches T_core_ is effective through evaporative cooling only (Sherwood and Huber, 2010). The laboratory data show that heat dissipation through evaporation, in a dry atmosphere, was effective up to T_a_ = 30°C only (Figures 1C,D). Above that, heat storage culminated in dangerously high T_sub_s (Figure 1B). Worrisomely, wild tarsiers are faced with constantly high relative humidity conditions which retard heat dissipation. A further concern is the argument that the rate of global warming has been greatly underestimated by the IPCC (IPCC, 2007; Rahmstorf et al., 2007; van Oldenborgh et al., 2009), and might be much higher than the feasible adaptive response of the species (Hughes, 2000; Root et al., 2003; Quintero and Wiens, 2013). Increases in the severity and frequency of extreme weather events, which are known to have devastating consequences (Boyles et al., 2011), have also been predicted (Meehl and Tebaldi, 2004; Jentsch et al., 2007; Luber and McGeehin, 2008). The future survival of wild *C. bancanus* is therefore highly questionable and supports the concerns expressed by Lovegrove et al. (2014a).

South East Asia is highly susceptible to the effects of El Niño-Southern Oscillation (ENSO). Thus, even though temperatures within the tropical belt may remain relatively stable, the region as a whole is highly unpredictable due to the effects of ENSO (Allan et al., 1996). As such, from an evolutionary perspective, the capacity for heterothermy, should tarsiers possess the ability, is likely to have been retained. Coincidently, there was a switch from El Niño to La Niña during the study, which would have resulted in a degree of climatic unpredictability. Given the shift in climate, the early morning cold snap on the 29th of June would have been an opportune time for them to enter torpor and they could have maximized the benefits by exploiting the daily increase in temperature to rewarm passively. Thus, based on the results of our study and those of previous work on tarsiers (Clarke, 1943; McNab and Wright, 1987; Lovegrove et al., 2014a), we conclude that heterothermy has yet to be observed in tarsiers. However, given the low sample size of free-ranging T_b_ data here and by Lovegrove et al. (2014a), we cannot rule out the potential for heterothermy within *C. bancanus* or *T. syrichta*, and certainly not for the rest of the Tarsiidae for which there are no data. That said, the apparent loss of heterothermy in tarsiers, as well as the lack of evidence for heterothermy in other haplorrhines, if true, suggests that adaptive heterothermy may have been lost either in the ancestor of the tarsiers or in the tarsier clade. We muse on our rationale below and identify also several potential haplorrhine primate species in which a search for heterothermy might fruitfully aid in resolving the primate adaptive-heterothermy phenotype.

Barring a few notable exceptions within the carnivores, monotremes and rodents, heterothermy is mostly observed in mammals smaller than 1 kg; mean weight of approximately 340 g (Geiser, 1998; Lovegrove, 2012a; Ruf and Geiser, 2015). Body size is an important consideration because thermal inertia in large-bodied animals hampers the reduction in T_b_ and thus the energetic benefits of heterothermy (Geiser, 1998; Ruf and Geiser, 2015). Observations of heterothermy in large and small sized animals suggests that they may even represent different conditions but more work on this topic is required (Geiser, 2001). Specifically within primates, heterothermic species range in M_b_ from the 30 g Madame Berthe's mouse lemur (*Microcebus berthae*; Dausmann and Warnecke, 2016) to the 350 g furry-eared dwarf lemur (*Cheirogaleus crossleyi*; Blanco and Rahalinarivo, 2010; Blanco et al., 2013), but the heaviest known primate heterotherm is the 400 g pygmy slow loris (*Nycticebus pygmaeus*; Ruf et al., 2015). No heterothermy was observed in the large 600 g sportive lemur (*Lepilemur ruficaudatus*; Schmid and Ganzhorn, 1996). Thus, even though body size may not be the only determining factor, it is an important consideration so we analysed the database of Isler et al. (2008) to identify other potential primate candidates that, based on body mass, could be heterotherms. The database contained the body mass values for 239 primates, 177 of which were haplorrhines. Excluding the tarsiers (Tarsiidae), there are 11 other haplorrhines that weigh 500 g or less and all belong to the family Cebidae namely *Callithrix pygmaea, C. jacchus, C. penicillate, C. argentata, C. humeralifera, C. aurita, Saguinus fuscicollis, S. niger, S. oedipus, S. nigricollis*, and *Callimico goeldii*. To the best of our knowledge, no evidence of torpor has been observed in these primates. However, although metabolic studies do exist for some (Boere et al., 2005; Kuehnel et al., 2012; Go et al., 2015; Ross et al., 2015; Lelegren et al., 2016), thermoregulatory studies with the potential to observe heterothermy have only been conducted for *C. jacchus* (Petry et al., 1986), *C. pygmaea* (Genoud et al., 1997), *S. oedipus* (Stonerook et al., 1994), and *C. goeldii* (Kälin et al., 2003; Power et al., 2003). While we cannot, at this point, discount the possibility of torpor in all Platyrrhini, the lack of torpor by *C. pygmaea*, the smallest and most eligible platyrrhine heterotherm, is congruent with our reasoning. We urge further investigation and suggest that future studies focus on the species listed here as they are the most likely to disprove our idea of the loss of heterothermy in non-Shrepsirrhini.

The reason(s) why adaptive heterothermy appears to have been lost in tarsiers at the Strepsirrhini-Haplorrhini split is unclear. However, it is worth exploring the similarities and dissimilarities between heterothermic members of the Strespirrhini and members of the Tarsiidae which might also explain the apparent loss of heterothermy in other haplorrhines. We share thoughts on factors directly related to energy expenditure namely, body size including the size of the brain, nocturnal habits, the effect of diet, reproduction and locomotion.

As discussed earlier, based on their M_b_, tarsiers should be eligible to express heterothermy. Thus, there may be another size-related factor that accounts for the lack of torpor use, such as brain size. Brain tissue is a metabolically expensive tissue. The increase in brain size of the haplorrhines and the consequent increase in metabolic demands reflected in higher BMRs (Isler and van Schaik, 2006) may have prohibited the use of heterothermy (Lovegrove, 2017). Tarsiers are nocturnal hunters that leap from tree to tree. The ability to successfully navigate, let alone hunt, in low light environments requires tremendous visual acuity, depth perception and neuronal accompaniment (Collins et al., 2005). Indeed, tarsiers display numerous specialized adaptations such as an enlarged lens, cornea and retina, as well as a high density of rods in the retina (>300,000 per mm^2^) in addition to neuronal adjustments, most notably enlarged primary and secondary visual cortex regions (Castenholz, 1984; Collins et al., 2005). However, these adaptations appear subject to a trade-off. Tarsier brains, relative to other primates, do not show an increase in mass, endocranial volume or encephalization quotient (Stephan, 1984; Grabowski et al., 2016). Instead, tarsiers compensate through a morphological readjustment of the brain by having enlarged occipital and temporal lobes and a diminished frontal lobe (Schwartz, 2003).

The Expensive-Tissue Hypothesis predicts that the metabolic costs associated with large brains in primates are off-set by reductions in the equally costly splanchnic tissue (Aiello and Wheeler, 1995). Thus, in addition to the morphological readjustment in tarsier brains, a further trade-off may be their dietary simplification and specialization. As previously mentioned, tarsiers are the only strict carnivorous primates, and it has been suggested that their dietary constriction may preclude the ability to employ heterothermy (Dausmann, 2008). However, the restricting factor may not be their diet *per se*, but rather the trade-off which their diet represents. An expansion of The Expensive-Tissue Hypothesis, the Expensive Brain Framework Hypothesis (Isler and van Schaik, 2009), proposed that the costs associated with relatively large brains must either accompany an increase in energy turnover or a reduction in energy expenditure; not limited to digestion. Within this framework, a portion of the energetic costs associated with a large primate brain and visual adaptations needed for their carnivorous nocturnal lifestyle (Crompton and Andau, 1986, 1987; Jablonski and Crompton, 1994) may be shared by a reduction in foetal growth rate, reproductive output and the cost of locomotion.

Tarsiers typically produce a single offspring and have gestation periods ranging between 157 and 180 days, longer than the gestation period of *Microcebus* spp. and *Cheirogaleus* spp., but comparable to those of *Galago* spp. and *Loris* spp. (Izard et al., 1985; Roberts, 1994). However, the M_b_ of tarsier neonates relative to their adult M_b_ as well as their neonate brain size is significantly larger than other primates (Roberts, 1994). The large neonatal brain size explains why, despite similar or even slower postnatal growth rates and weaning periods relative to other primates, tarsier infants have extremely rapid behavioural development, particularly in foraging behaviour (Roberts, 1994). As such, development factors, in combination with dietary constraints may preclude heterothermy. Furthermore, in *C. bancanus* at least, homeothermy may be related to the fact that they remain reproductively active throughout the year (MacKinnon and MacKinnon, 1980; Wright et al., 1986). Males thus continuously produce sperm and a greater variation in body temperature may compromise sperm viability as spermatogenesis, sperm storage and sperm maturation processes are optimized at 34–36°C (Lovegrove, 2014).

With regards to the cost of locomotion, irrespective of the mode of locomotion the energetic investment is proportional to M_b_ (Schmidt-Nielsen, 1997; Withers et al., 2016). Of the various modes of locomotion, saltation, while at low speed, is known to be energetically more costly than typical walking. However, it becomes energetically more efficient as the speed increases (Withers et al., 2016). Tarsiers do not typically hop around on the ground at low speed, but leap fleetly around in trees. In comparison to other saltatorial primates, tarsiers seemingly have the lowest energetic investment (Warren and Crompton, 1998). Warren and Crompton (1998) considered the kinetic cost of leaping, M_b_, distant travelled, home range size and metabolism, and showed that even though tarsiers travelled the farthest and had the largest home range of all their study species, they had the lowest absolute and relative cost of locomotion. This reduction in energetic expense presumably stems from the tarsiers' musculo-skeletal anatomical adaptations (Jouffroy et al., 1984; Peters and Preuschoft, 1984; Schultz, 1984; Anemone and Nachman, 2003); the most obvious of which is the eponymous elongated tarsal bones.

## Author contributions

SW and BL conceived and designed the study. SW performed the data collection, analyses and drafted the manuscript. BL contributed to and approved the manuscript. AT secured capture permits, provided logistical support, as well as approved the manuscript.

### Conflict of interest statement

The authors declare that the research was conducted in the absence of any commercial or financial relationships that could be construed as a potential conflict of interest.
